# Antimicrobial Drug Resistance: "Prediction Is Very Difficult, Especially about the Future"^1^

**DOI:** 10.3201/eid1110.051014

**Published:** 2005-10

**Authors:** Patrice Courvalin

**Affiliations:** * Institut Pasteur, Paris, France

**Keywords:** antimicrobial drugs, antibiotics, resistance, evolution

## Abstract

Evolution of bacteria towards resistance to antimicrobial drugs, including multidrug resistance, is unavoidable because it represents a particular aspect of the general evolution of bacteria that is unstoppable. Therefore, the only means of dealing with this situation is to delay the emergence and subsequent dissemination of resistant bacteria or resistance genes. Resistance to antimicrobial drugs in bacteria can result from mutations in housekeeping structural or regulatory genes. Alternatively, resistance can result from the horizontal acquisition of foreign genetic information. The 2 phenomena are not mutually exclusive and can be associated in the emergence and more efficient spread of resistance. This review discusses the predictable future of the relationship between antimicrobial drugs and bacteria.

## Abstract 2

Although resistance cannot be prevented, much can be done to delay it.

Over the last 60 years, bacteria and, in particular, those pathogenic for humans have evolved toward antimicrobial drug resistance. This evolution has 2 key steps: emergence and dissemination of resistance.

Humans cannot affect emergence because it occurs by chance and represents a particular aspect of bacterial evolution. Emergence can result from mutations in housekeeping structural or regulatory genes or from acquiring foreign genetic information. However, much can be done to delay the subsequent spread of resistance. Dissemination can occur at the level of the bacteria (clonal spread), replicons (plasmid epidemics), or of the genes (transposons). These 3 levels of dissemination, which coexist in nature, are not only infectious but also exponential, since all are associated with DNA duplication. Clonal dissemination is associated with chromosome replication, plasmid conjugation with replicative transfer, and gene migration with replicative transposition (*1*). The spread of resistance has repeatedly been shown to be associated with antimicrobial drug use (*2*), which stresses the importance of the prudent use of these drugs; a notion reinforced by the observation that resistance is slowly reversible (*3**,**4*).

Therefore, attempting to predict the future of the relationship between antimicrobial drugs and bacteria is conceptually challenging and potentially useful. For the sake of convenience, the examples will be taken mainly from the work carried out in the author’s laboratory, although numerous other examples can be found in the literature.

The clinically relevant predictable resistance types are listed in the Table. Although they have not yet been reported, they may exist in nature; their apparent absence is, at least for some of them, rather surprising. For example, streptococci, including pneumococci and groups A, C, and G, can easily acquire in vitro conjugative plasmids from enterococci and stably maintain and phenotypically express them (*5*). Therefore, it is all the more surprising that genes commonly found on plasmids in the latter bacterial genus, such as *bla* for penicillinase production and *aac6*´-*aph2*´´ for resistance to nearly all commercially available aminoglycosides, have not yet emerged in streptococci. The situation is even more unusual for *Listeria* spp., which remain susceptible to most antimicrobial drugs even though they can acquire plasmids from both enterococci and staphylococci (*6*). However, the obligate intracellular existence of *Chlamydia* spp. likely protects them from contact with foreign DNA and accounts for their retained susceptibility to antimicrobial drugs.

**Table:** Predictable resistance types

Organism	Resistance phenotype or mechanism
*Streptococcus pneumoniae*	Penicillinase, gentamicin, glycopeptides
*Streptococcus* groups A, C, G	Penicillins
*Listeria monocytogenes*	Penicillins, gentamicin
*Legionella pneumophila*	Macrolides, fluoroquinolones
*Salmonella enterica* serovar Typhi	Third-generation cephalosporins
*Haemophilus influenzae*	Third-generation cephalosporins
*Neisseria meningitidis*	Third-generation cephalosporins
*Brucella* spp.	Tetracyclines, rifampin, streptomycin
*Clostridium difficile*	Glycopeptides
*C*. *perfringens*	Penicillinase
*Chlamydia* spp.	Tetracyclines

## How To Anticipate Resistance

**Figure 1:**
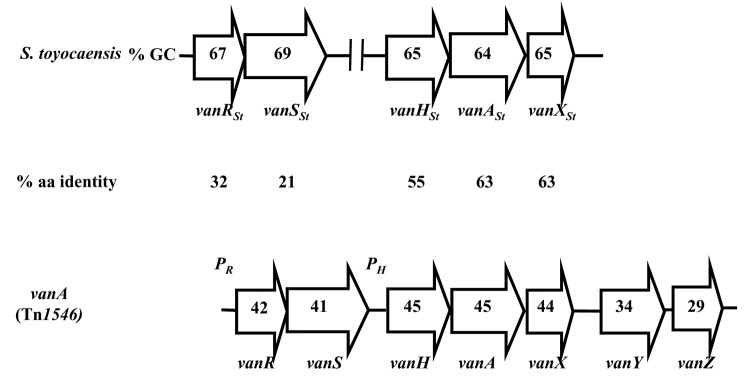
Comparison of the van gene cluster from the glycopeptide producer Streptomyces toyocaensis (*32*) and of the vanA operon (*33*) from gram-positive cocci. Open arrows represent coding sequences and indicate direction of transcription. The guanosine plus cytosine content (% GC) is indicated in the arrows. The percentage of amino acid (aa) identity between the deduced proteins is indicated under the arrows.

One should distinguish "natural" antimicrobial drugs (e.g., kanamycin), which are produced by microorganisms from the environment, from semisynthetic (e.g., amikacin) and entirely synthetic compounds (e.g., quinolones), which are produced, at least in part, by humans. The microorganisms that produce natural antimicrobial drugs have to protect themselves from the products of their own secondary metabolism. To ensure their survival, these organisms have developed self-protection mechanisms similar to those found in resistant human pathogens (*7*); this observation led to the idea that the producers constitute the pool of origin of certain resistance genes (*8*). Therefore, the study of resistance in the strain used for the industrial production of an antimicrobial agent could allow a strong prediction about the mechanism that will be found later in bacteria pathogenic for humans. For example, the study of glycopeptide producers would have allowed the elucidation, long before it actually occurred, of the mechanism by which enterococci and, more recently, staphylococci could become resistant to these drugs (Figure 1).

As already noted, bacteria are resistant to antimicrobial drugs after horizontal DNA transfer or mutations. Thus, another prediction that can be made is that bacteria will transfer to susceptible species, resistance determinants already known in other bacterial genera, for example, the recent acquisition of glycopeptide resistance by *Staphylococcus aureus* from *Enterococcus* spp (*9*). However, this prediction is limited since it refers to mechanisms that have already been explained. In addition to being antimicrobial agent producers, the commensal bacteria of mammals, particularly those in the gut, also represent a pool of origin for resistance genes. When infections are treated with an antimicrobial agent, all bacteria in the host are affected, including the commensal flora, which could result in the selection of resistant commensals, particularly in children who are administered oral antimicrobial drugs too frequently. Large numbers of these resident bacteria are present in the digestive tract where they are often in transient, but intimate, contact with exogenous microorganisms that are in various developmental states, including competence. These conditions favor the transfer of genes by transformation and by conjugation. Including antimicrobial drugs in animal feed also leads to the selection of a pool of resistance genes that can be transferred to commensal bacteria in the human digestive tract and thus ultimately to human pathogens, even when selective pressure is absent (*10*).

In the case of mutations, predictions can be supported by 2 types of experimental approaches: in vivo with intact bacteria or in vitro by using DNA. Mutations resulting in resistance can be obtained in an accelerated fashion by using hypermutators, that is, bacteria deficient in the DNA repair system (*11*). Mutations are also accumulated by using continuous cultures, preferably in chemostats under suitable selective pressure. A similar enhanced rate of evolution can be obtained by (saturated) DNA mutagenesis, followed by transformation into an appropriate host. This technique, for example, was used successfully to study the extent of variations in penicillinase genes that generate extended-spectrum β-lactamase agents (*12*).

## Pathways to Resistance

### Modulation of Gene Expression

In addition to developing mutations in structural genes for drug targets, bacteria can become resistant after mutational events in motifs for gene expression, such as promoters (*13*), in regulatory modules, such as 2-component regulatory systems (*14*), or positioning upstream from a gene of a mobile (*15**,**16*) or stable (*17*) promoter. Enhanced expression of genetic information can also be caused by alterations in translation attenuation (*18*). The DNA regions involved in gene regulation are not always adjacent to the target gene. This factor makes finding regulatory mutations more complicated and makes detecting resistance by this mechanism generally impossible by genotypic techniques (*19*).

### Dissemination by Transformation

Dissemination by transformation is more likely in spontaneously transformable bacterial species such as *Streptococcus pneumoniae*, *Acinetobacter* spp., and *Neisseria* spp. These bacteria can easily acquire, integrate, and express stretches of DNA. Since the latter can include portions of foreign chromosomes, this process renders chromosomal mutations infectious (*20*).

### Combination of Mechanisms

Because of increased activity or the expanded spectrum of certain drug classes (e.g., β-lactam agents and fluoroquinolones) or of local therapy (e.g., extremely high concentrations in the gut after oral administration of glycopeptides that do not cross the digestive barrier) bacteria need to combine mechanisms that confer resistance to the same class of molecules. This process is necessary to achieve high-level resistance (*21*) or expand the substrate range provided by a single resistance mechanism (*22*). An example is provided by gram-negative bacteria and β-lactam agents. Extended-spectrum β-lactamase agents are point mutants of "old" penicillinases (*23*). Generally, the biologic price to pay for extending the substrate range of this enzyme is hypersusceptibility to β-lactamase inhibitors. However, the presence in certain enterobacteria of the gene for a penicillinase on a small multicopy plasmid, which results in production of large amounts of the enzyme and confers resistance to β-lactamase inhibitors by trapping (*24*). The net result of this combinatorial approach is the production of gram-negative bacteria that are resistant to all β-lactam agents, except carbapenems and cephamycins, which are not substrates for the enzymes.

### Two Mechanisms Involved in Resistance Are Increasingly Frequent

#### Impermeability

No antimicrobial agent is active against all bacteria. In fact, the intrinsic (natural) resistance of bacteria, which is better designated as insensitivity, defines the spectrum of activity of a drug, usually because the antimicrobial drug does not penetrate the bacteria. However, microorganisms can become resistant to nearly all drug classes, including those that act at the surface of the bacteria (e.g., β-lactam agents, bacitracin), by impermeability. This resistance can be secondary to 2 distinct pathways: passive, which involves alterations of outer membrane proteins, the porins, which decrease the rate of entry of antimicrobial drugs into the bacteria by diminution of the pore size (*25*), and active, which involves overexpression of an indigenous efflux pump that exports the antimicrobial drug outside the cell after a regulatory mutation (*26*).

#### Trapping

**Figure 2:**
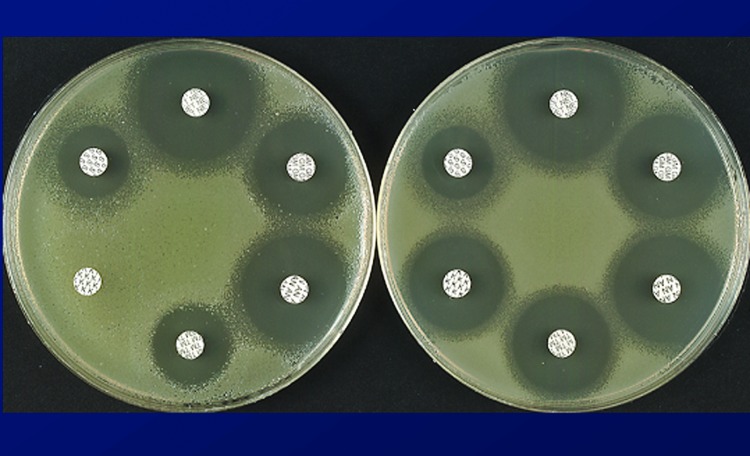
Disk susceptibility test results of Escherichia coli BM694 (left) and of strain BM694 harboring plasmid pAT346, which confers tobramycin resistance by trapping (right) (*27*).

The mechanism of trapping, already mentioned in the case of resistance to β-lactam agents by a combination of β-lactamases, allows titration of the drugs, an alternative to impermeability, for lowering the intracellular concentrations of the antimicrobial drugs. This mechanism also works against aminoglycosides in bacteria that overproduce an enzyme that has affinity for a drug they cannot inactivate since it lacks the modification site (Figure 2) (*27**,**28*). This mechanism has also been proposed to account for low-level resistance to glycopeptides in staphylococci by overproducing target sites in the outer layers of the peptidoglycan; thus, the antimicrobial drug does not reach the important target sites where the wall is assembled on the outer surface of the cytoplasmic membrane (*29*).

## Prediction at the Genetic Level

Genes from gram-positive cocci can be transferred by conjugation (of plasmids or transposons) not only among these microorganisms but also to gram-negative bacteria (*30*). The reverse is not true because of limitations in heterologous gene expression. Consequently, one can confidently predict further dissemination of the resistance gene pool of gram-positive to gram-negative bacteria.

We have been aware for a long period that "everything that exists in the universe is the result of chance and necessity" (Democritus, 460–370 BC), which holds true for antimicrobial drug resistance. Most unfortunately, and for various reasons, it is extremely difficult to think like a bacterium. In other words, predicting the emergence of resistance to a drug class by a precise molecular mechanism is nearly impossible (e.g., glycopeptide resistance in enterococci or plasmid-mediated resistance to fluoroquinolones). We also cannot anticipate, among all the conceivable mechanisms of resistance (*31*), which will emerge first under natural conditions. However, based on the understanding during recent decades of the physiology (genetics and biochemistry) of bacterial resistance to antimicrobial drugs, impressive progress has been made in the techniques for in vitro detection and for elucidation of resistance. This progress should, in turn, be helpful in delaying the second step of resistance: dissemination.
